# Novel predators emit novel cues: a mechanism for prey naivety towards alien predators

**DOI:** 10.1038/s41598-017-16656-z

**Published:** 2017-11-27

**Authors:** Alexandra J. R. Carthey, Martin P. Bucknall, Kaja Wierucka, Peter B. Banks

**Affiliations:** 1 Department of Biological Sciences, Macquarie University, Sydney, 2109 Australia; 2 Bioanalytical Mass Spectrometry Facility, Mark Wainwright Analytical Centre, The University of New South Wales, Sydney, 2052 Australia; 3 Institut des Neurosciences Paris-Saclay, Université Paris-Saclay, CNRS (UMR 9197), Université Paris-Sud, Orsay, 91405 France; 4 School of Life and Environmental Sciences, The University of Sydney, Sydney, 2006 Australia

## Abstract

Detecting enemies is crucial for survival and a trait that develops over an evolutionary timeframe. Introduced species disrupt coevolved systems of communication and detection in their new ranges, often leading to devastating impacts. The classic example is prey naivety towards alien predators, whereby prey fail to recognise a new predator. Yet exactly why native prey fail to recognise alien predators remains puzzling. Naivety theory predicts that it is because novel predators emit novel cues. Distantly related animals have distinct evolutionary histories, physiologies and ecologies, predicting they will emit different cues. Yet it also possible that all predators emit similar cues because they are carnivorous. We investigate whether odour cues differ between placental and marsupial carnivores in Australia, where native prey experienced only marsupial mammal predation until ~4000 years ago. We compared volatile chemical profiles of urine, scats and bedding from four placental and three marsupial predators. Chemical profiles showed little overlap between placental and marsupial carnivores across all odour types, suggesting that cue novelty is a plausible mechanism for prey naivety towards alien predators. Our results also suggest a role for olfactory cues to complement visual appearance and vocalisations as biologically meaningful ways to differentiate species.

## Introduction

The ability to detect and recognise predators is crucial for survival, and is learned or evolved over long periods of shared evolutionary history^1,2^. The introduction of species beyond their native ranges leads to the disruption of coevolved detection and communication systems^3,4^, leading in many cases to devastating impacts^5,6^. Understanding biotic interactions between alien and native species can help to predict not only invader impacts, but also the establishment and spread of invasive species^7,8^. Alien predators have twice the impact that native predators have on native prey^9^, a phenomenon thought to be driven by prey naivety, or the failure to recognise an alien predator and respond accordingly^10,11^. There is a large body of research into native prey naivety towards alien predators, which is usually experimentally assessed as a failure to recognise alien predators’ olfactory (e.g.^12–15^), visual (e.g.^16^) or acoustic cues (e.g.^17^). Native prey naivety has been observed in many invaded environments^11^. But why do native prey fail to recognise alien predators? Naivety theory is predicated on the idea that novel predators emit novel cues^18–21^. Yet to date, this key prediction remains untested. Olfactory predator recognition is a powerful system in which to test this idea, because most mammals and many vertebrates have a highly developed olfactory system and use the information contained in odour cues to recognise and avoid their predators^22^, among many other varied and important functions^23^. While many studies have demonstrated a lack of response to alien predator odours in native prey (e.g.^12–15^), the critical question of whether distantly related mammalian carnivores produce different odours remains unanswered.

In the broadest sense, a cue is sensory information available to the prey that indicates the presence, or the potential presence, of the predator. For an animal to respond to a cue, both perceptual and cognitive processes are required. Detection theory^24^ posits that there are two components underpinning a response. The first is cue discriminability, or how well the animal can identify the cue, which necessarily involves the sensory and perceptual systems. The second component is the animal’s response bias, which reflects the relative costs of under-responding *versus* over-responding to the cue. The prey’s perceptual and cognitive processes, as well as their response bias, are shaped by the prey’s evolutionary experience with native predators and their cues^6,24^. Thus, alien predators that are sufficiently novel may circumvent the detection processes of native prey, leading to naivety and its attendant consequences^11,18,19,24^. Naivety, therefore, is fundamentally a case of evolutionary mismatch between a novel predator’s cues and the cue detection and response process of prey^3^.

Australia has the worst extinction record globally for small and medium-sized native mammals^6^, a fact that has been attributed to prey naivety towards introduced dogs (*Canis lupus familiaris*), foxes (*Vulpes vulpes*), and cats (*Felis catus*), all introduced by humans some 150 years ago^10,25^. Each of these alien predators are placental mammals (Mammalia: Placentalia^26^), whereas Australia’s native mammalian predators are all marsupial (Mammalia: Marsupialia^26^). The one exception is the placental dingo (*Canis lupus dingo*), which arrived on the continent approximately 4000 years ago^27^. Placental and marsupial mammals diverged ~160 million years ago^28^, meaning they are very distantly related taxa. Wild dogs, cats, and foxes are widespread across mainland Australia, with the exception that foxes do not extend to the tropical far north of the continent^26^. Dogs, dingoes, and foxes all belong to the Canidae, meaning they are more closely related to each other than they are to cats (Felidae). Australia’s largest marsupial predators include quolls (*Dasyurus spp*.) and Tasmanian devils (*Sarcophilus harrisii*), which are each other’s’ closest living relatives – they are much more closely related to one another than they are to any of the placental predators^29^.

Cox and Lima^19^ proposed that prey will be naïve to an alien predator if it represents a novel predator archetype, defining an archetype as “the set of predators against which a given suite of antipredator adaptations is effective”, and suggesting predator families as a practical proxy for different archetypes^19^. Given the extensive evolutionary distance between placental and marsupial predators, the Australian scenario provides an unrivalled opportunity to test the hypothesis that novel predator archetypes emit novel cues (the “cue similarity hypothesis”^6,24^). If placental predators emit fundamentally different cues to native marsupial predators, it would provide a mechanistic explanation for prey naivety in Australia: an evolutionary mismatch between the sensory and perceptual abilities of native prey and the novel cues of alien predators.

Olfactory cues work in the dark and linger after the donor animal has departed, allowing communication *in absentia* ^30^. Many mammals use olfactory cues to detect and avoid their predators^22,30^, but olfactory cues can also convey information about the donor’s sex, age, social status, sexual receptivity, and territory ownership^31^, and are known to be involved in many social contexts including mating behaviour^32^. In order to function in these ecological roles, mammalian chemical cues must allow for recognition, and we would therefore expect them to differ among groups of individuals^33,34^. Social cues must differ sufficiently among species to maintain reproductive isolation and enable both recognition of conspecifics and mediation of interspecific communication within communities^35,36^. Indeed, phylogenetic distances have been correlated with differences in olfactory cues for both felids^37^ and lemurs^38^. For these reasons, marsupial and placental mammals seem likely to produce different olfactory cues. We would also expect that native prey experienced with marsupial predators would have sensory and perceptual capabilities attuned to the chemical components of marsupial predators – not to those of placentals^3,4,24^. In a similar way, Australian native anuran tadpoles are thought to speak a different chemical “language” to invasive cane toad tadpoles (*Bufo marinus*), which can be lured and trapped with toad-specific chemical cues, that are ignored by native frogs^39^.

On the other hand, predator odours are thought to share chemical components resulting from meat metabolism – a predator “leitmotif” or “generalised meat-eater cue” that labels even unfamiliar predators as dangerous to prey, without requiring prior experience^40–42^. A compound common to several placental mammal urines, 2-Phenylethylamine (2-PEA), has been shown to bind to a specific odour receptor and trigger innate avoidance behaviours in rats and mice^43^. 2-PEA is enriched in several placental carnivore species’ urines, but undetectable or in very low concentrations in many placental herbivore urines. The authors suggest it is “a major, but not exclusive, component of predator urine that is recognised by the olfactory system” of prey. However, there are no available data on the presence of 2-PEA in marsupial mammal orders, or in faecal or body/fur odours of placental or marsupial mammals. It is a similar story for 3,3-dimethyl-1,2-dithiolane (DMDIT), 2,4,5-trimethyl-thiazoline (TMT), and several other compounds commonly fractionated out of fox, cat and mustelid urine and scats in the hopes of identifying a singular compound that indicates that an odour cue is predator-derived^44^. Experimental evidence for any single component that labels a cue as predator-derived is lacking – none of these compounds have managed to frighten all prey animals tested with them^44^. In fact, it is now clear that the varying ratios and concentrations of the different compounds within a mix change the meaning of odour cues significantly^45^. It is also likely that different compounds are important for predator recognition through different odour types (such as urine, scats, and body/fur), and within distantly related mammalian orders such as the marsupials^46^. In general, individual odorous compounds are not sufficient to elicit antipredator responses in prey – rather, prey appear to learn or evolve the ability to recognise “signature mixes” of compounds associated with particular predators^32,43,46^.

Despite the importance of olfactory communication in mammals, research into the chemical composition of these olfactory cues has progressed relatively slowly^47^. This is partly due to the highly complex nature of the mammalian olfactory communication system^45^. While olfaction and chemical signalling in invertebrates is well-understood, and invertebrate responses to particular compounds can be measured via electroantennogram^48^, research into mammalian chemical signalling relies on prohibitively cumbersome bio-behavioural assays^32,45,47,49^. Widely variable concentrations and high background noise are typical of mammalian secretions and excretions, making the search for mammalian chemical signals an ongoing challenge^50^. Nevertheless, recent advances in bioanalytical chemistry techniques make it possible to create profiles of the volatile compounds in the headspace above samples, giving insight into the “signature mixture” that would be available as olfactory information to another animal. Importantly, the goal is not to identify chemical signals^44,51^, but rather to make broad-level comparisons of the presence or absence, and relative amounts, of volatile compounds (the chemical profile) in samples from different species and higher taxonomic groups (e.g.^52^). Here we aim to determine whether the “signature mixes” of volatile compounds making up different species’ or marsupial and placental mammals’ chemical profiles are similar, or distinct.

We compare the volatile chemical profiles of scat, urine and bedding (intended to capture integumentary or “skin/fur” odour) samples from four placental (dingoes, dogs, foxes and cats) and three marsupial (tiger quoll *D. maculatus*, eastern quoll *D. viverrinus*, and Tasmanian devil) predators of small and medium sized Australian mammal prey. Naivety of native prey to these placental predators’ cues has been well-documented^10^ – for example, bush rats (*Rattus fuscipes*) do not avoid dog faecal odour^14,15^, tammar wallabies (*Macropus eugenii*) and red-necked pademelons (*Thylogale thetis*) ignore red fox faecal odour^12^, and bettongs (*Bettongia lesueur*) do not respond to red fox models^13^. We predicted that placental carnivore odour profiles would differ significantly and substantially from those of marsupial carnivores. As odours play such an important social communication role for each of these taxa, we also predicted that odour profiles would differ significantly among the individual predator species, reflecting the evolution of divergent communication systems along distinct evolutionary pathways.

## Materials and Methods

### Sample collection

We collected scat, urine and body odour samples from dingoes, dogs, cats, foxes, tiger quolls, and Tasmanian devils. We collected opportunistic urine samples from eastern quolls, but not scat or body odour samples. All three types of odour have induced responses in coevolved prey in previous experimental work^31^. Bedding samples captured the integumentary odours of predators, including skin/fur, salivary and other gland secretions.

Scat samples were collected opportunely by shelter, sanctuary or zoo staff, or by pet owners, within 12 hours of being deposited, and frozen at −20 °C. Urine samples were caught on voiding with a funnel and jar, syringed from concrete enclosure floors immediately after voiding (some Tasmanian devil samples), collected by animal shelter staff during routine desexing operations (some cat and some dog samples), or collected from freshly shot carcasses (some fox samples). For bedding samples, we washed (without detergent) and air-dried beige cotton towels (65 × 135 cm) to remove residual manufacturing odours. A single towel was then placed amongst the bedding of an individual animal and left for 14 nights. Control bedding samples were taken from towels that were treated identically to sample towels, but were not placed with an animal. Instead, they were left hanging on a rack in an undercover outdoor area for 14 nights. Sample size ranged from 3 to 9 per odour type, per species (see Supplementary Tables S2–S4 for exact number of predator individuals sampled for each species and odour type).

Each predator species has specific dietary requirements, and we collected ~200 samples from multiple owners, sanctuaries, zoological parks, rescue shelters, and wild-shot carcasses, making it infeasible to manipulate predator diets. However, captive animals were fed a natural diet in zoological parks and sanctuaries, domestic pets were fed commercial pet food and occasional additional raw meats, and wild-shot foxes consumed wild diets. Previous work suggesting that predator diet influences prey responses to predator odours involves prey recognising when predators have consumed conspecifics^53^, or when carnivorous predators have been fed a vegetarian diet^42^. The comparatively small differences in the make-up of the natural carnivorous diets of the predators tested here were therefore not thought to be problematic. See Supplementary Tables S2–S4 for detailed information on sample sizes and the source of each sample.

Samples were stored in inert borosilicate glass bottles with polypropylene lids (Schott or Boeco brand), frozen immediately at −20 °C, and then transferred to −80 °C until use (a maximum of two months). Storage bottles were baked at 130 °C for a minimum of two hours before use to remove any residues or contaminants. All samples were handled with disposable gloves to prevent cross-contamination.

### Laboratory methods

We chose solid phase microextraction (SPME) gas chromatography-mass spectrometry (GC-MS) to sample and analyse the volatiles in the headspace above the urine, scats and bedding samples for the following reasons: 1) SPME GC-MS is commonly used to investigate olfactory properties of biological substances^54^, 2) detection limits are similar between mammalian noses and the SPME GC-MS methodology for the volatile compounds likely to be found in mammalian secretions and excretions^54^, 3) SPME fiber coatings enable headspace sampling without the use of solvents or other additional sample preparation steps that would further remove our methodology from the process of a mammalian nose sampling volatiles in the field^50,54^.

Samples were defrosted at room temperature (~22 °C) immediately prior to analysis and measured into 20 ml glass headspace vials with Teflon septa (Thermo Fisher Scientific) that had previously been baked at 130 °C for a minimum of two hours. An aliquot (1 mL) of urine, or 3 g of either towel or scat was placed in each vial. Fluorobenzene (C_6_H_5_F) was added to each sample in aqueous solution (0.5 μg fluorobenzene to body odour samples; 5 μg to urine and scat samples) as an internal standard. Fluorobenzene was chosen as an internal standard because it has similar volatility and molecular weight to the potential study compounds, is unlikely to react with the study compounds or sample matrices, is not an erogenous mammalian occurring compound and should not therefore be in the samples already. Control samples were also prepared: 3 ml Milli-Q water (Merck Millipore; Germany) with 0.5 µg fluorobenzene as a control for scats, 1 mL Milli-Q water with 5 µg fluorobenzene as a control for urine, or 3 g clean towel with 5 µg fluorobenzene as a control for body odour.

A 65 μm polydimethylsiloxane/divinylbenzene SPME fibre was exposed to the headspace above the sample for 20 minutes at ambient temperature (22 °C). SPME GC-MS were performed using a Thermo Trace DSQ II GC-MS coupled with a Thermo TriPlus HS autosampler (Thermo Scientific, Watham, MA). The inlet port temperature was 230 °C, operated in splitless mode with a 1 mm glass liner. The fibre was desorbed in the inlet port for 2 minutes, then purged with carrier gas in the inlet at a split flow of 150 mL/min for 15 minutes, to condition it for the next analysis. The GC was fitted with a mid-polarity free fatty acids phase column (HP-FFAP, 50 m × 0.2 mm, 0.3 µm film thickness, Agilent Technologies) and Helium was used as the carrier gas (0.8 mL/min). The oven temperature program was 40 °C for 3 minutes, then 5 °C per minute increase to 60 °C, then 3.5 °C per minute to 120 °C, 5 °C per minute to 180 °C, and then 10 °C per minute to a final temperature of 240 °C, where it was held for 10 minutes. The mass spectrometer was operated in electron impact ionization (EI+) mode, the ion source was set at 200 °C and the GC transfer line to 240 °C. Mass spectra were acquired over the range m/z 35–550 at a rate of 2.5 scans per second. Optimal adsorption and desorption conditions were determined by analysing surplus scat samples.

### Data processing and statistical analysis

Peaks were manually integrated on chromatograms using the Qual Browser module of Thermo XCalibur version 2.1.0 mass spectrometry software, based on mass spectra and the retention time of each integrated peak relative to fluorobenzene. Both peak detection and intensity relative to fluorobenzene were recorded. Compounds found in control samples (clean towel or Milli-Q water) were excluded from further analysis. Peak identities were determined by comparison of their averaged, baseline subtracted mass spectra with those contained in the NIST 2011 / Wiley 9 Combined Library of Mass Spectra using NIST MS Search Version 2.0 g R1 software. Peak matches with a score greater than 900/1000 were considered valid.

Statistical analyses were carried out in Primer v6^55^. Following a fourth root transformation of the dataset, we ran one-way ANOSIM analyses (based on a Bray-Curtis index), to look for differences in the presence and relative abundance of chemical compounds between marsupial and placental predators, and among the predator species sampled. We conducted pairwise comparisons to further investigate species differences. A nonmetric multidimensional scaling (nMDS) plot was used to visualize differences between tested groups (created using package VEGAN^56^ in R 3.2.2^57^). Similarity percentage analyses (SIMPER^55^) were conducted to determine whether differences among groups were determined by a few or multiple compounds. Finally, PERMDISP was run for each analysis (as recommended by Clarke & Gorley^58^ as an accompaniment to ANOSIM procedures), to confirm that the different groups had similar levels of dispersion to one another – i.e. any differences found were based on differences of group location rather than differences in group dispersion.

All data generated and analysed during this study are included in the Supplementary Information files.

## Results

**Figure 1:**
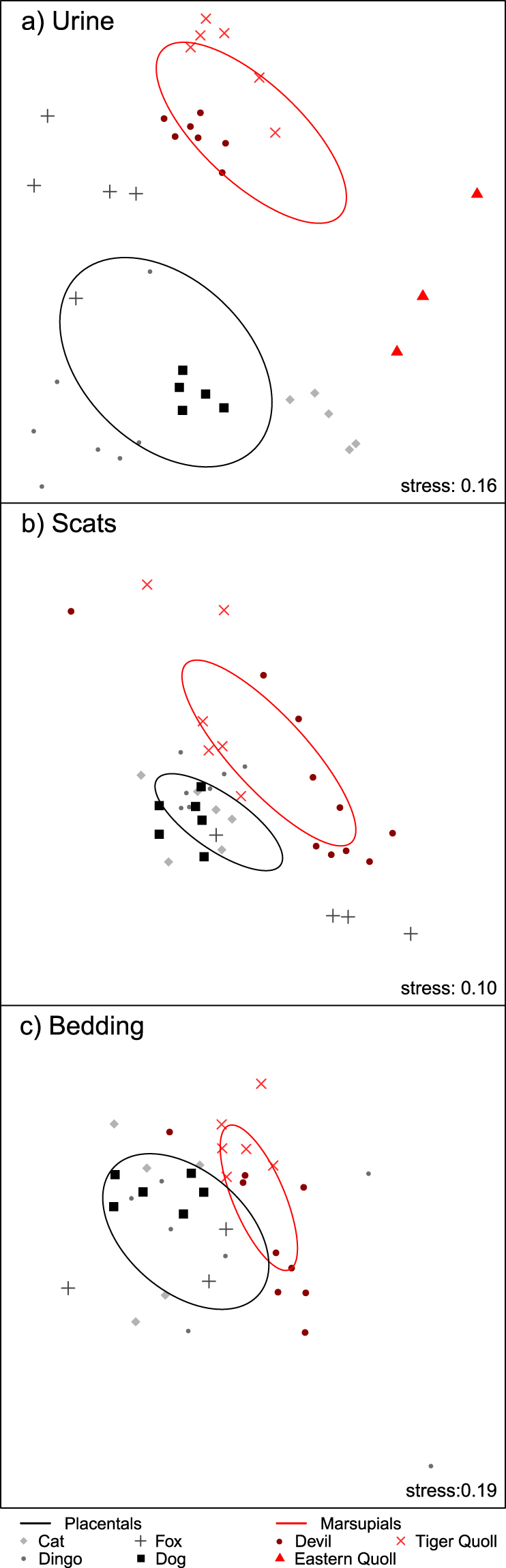
Visualisation of differences in chemical profiles of marsupial and eutherian predators’ odours for (**a**) scat, (**b**) urine and (**c**) bedding samples. Points represent samples, and the distances between points represent the degree of similarity: points closer together are more similar. Dispersion ellipses were drawn using standard deviation of point scores, with the weighted correlation defining the direction of the principal axis of the ellipse.

The chemical composition of marsupial predator odours differed significantly to that of placental predator odours across all three odour sample types, with urine samples showing the most pronounced differences (Fig. 1; urine: R = 0.536, *p* < 0.001; scats: R = 0.365, *p* = 0.002; bedding: R = 0.243, *p* = 0.003). Odour profiles also differed among individual predator species for urine (R = 0.921, *p* < 0.001), scats (R = 0.456, *p* < 0.001), and bedding samples (R = 0.395, *p* < 0.001), with differences again being most pronounced for urine samples. The chemical profile of each predator species’ urine was different from every other species, except for dingoes and dogs (Fig. 1, Table 1). Each of the predator species’ scat odour profiles differed from every other predator species, except for dingoes and dogs, and devils and foxes (Fig. 1, Table 1). Finally, marsupial (tiger quoll and devil) bedding samples were different from one another and from all placental predator bedding samples. The placental predator bedding odours did not differ from one another, with the exception of dogs and foxes (Fig. 1, Table 1). Differences among species and between marsupial and placental mammals were not driven by the presence or absence of a few dominant compounds, but rather by the varying mixture of numerous compounds across samples and among groups (SIMPER results, Supplementary Table S1). No single compound contributed to more than 2.6% of the differences observed between any two groups, and fifty percent of the observed differences between marsupials and placentals were explained by 52, 72 and 42 compounds in urine, scat and bedding samples, respectively (SIMPER, Supplementary Table S1).

**Table 1:**
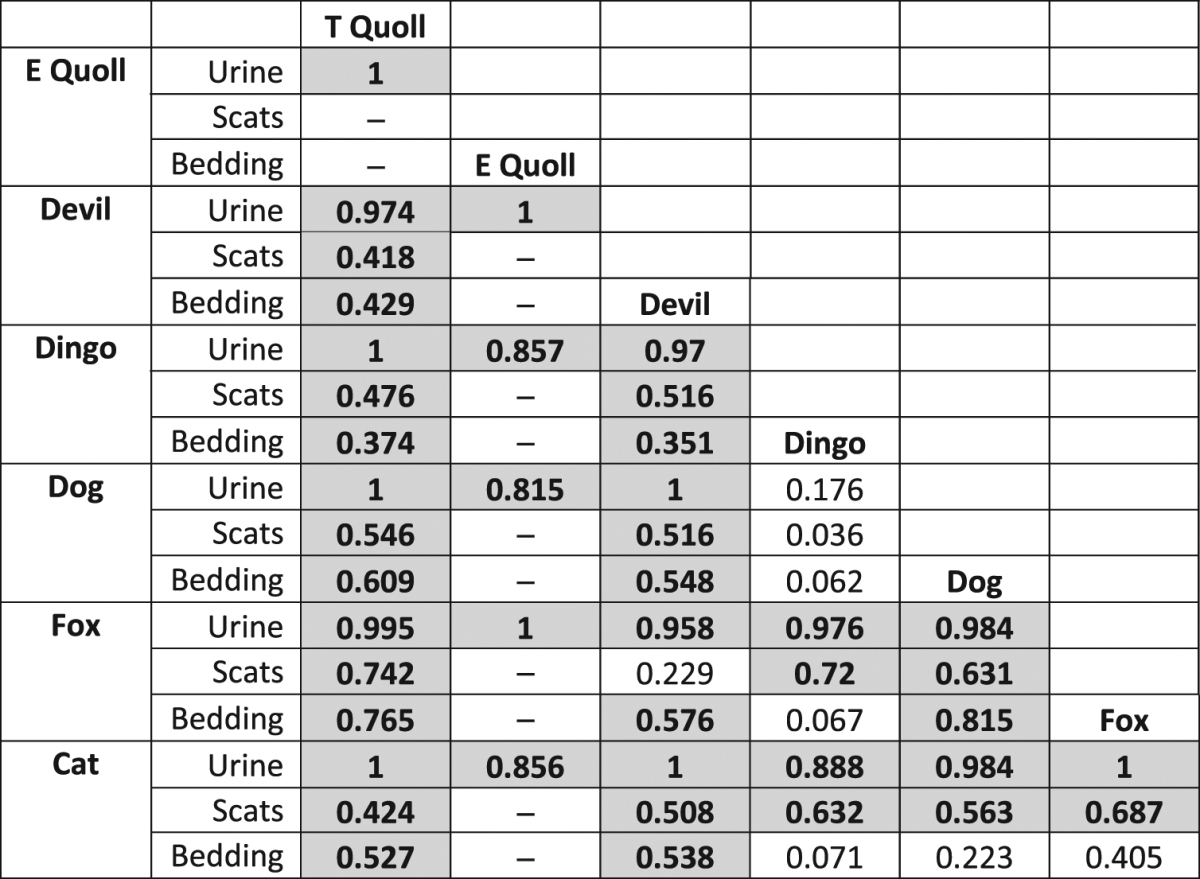
ANOSIM pairwise comparisons among predator species’ chemical profiles. Interpretation of R values are advised over Bonferroni-type adjustments of *p* values for ANOSIM pairwise comparisons^58^, hence R values and significance based on unadjusted *p* values are reported. Significant differences between predator species’ chemical profiles (*p* < 0.05) are marked with bold text and a grey background. R values closer to 1 indicate a greater dissimilarity between groups, and *vice versa* for R values closer to 0.

## Discussion

The volatile chemical profiles of placental carnivore urine, scats and bedding were all significantly different to those of the marsupial carnivores (Table 1), with each of these taxonomic groups separating out clearly on the nMDS plots (Fig. 1). Thus, in invaded Australian ecosystems, alien predators do emit fundamentally different information in their odour profiles in comparison to native predators. The idea that alien predators emit novel cues, and that this results in prey failing to recognise and defend against predators, is critical to the prey naivety hypothesis^18,19^. Failure to recognise a predator’s cues precludes any kind of later antipredator response, leading to high mortality, declines, and potentially extinctions^6,18,19^. This process has been repeatedly linked with Australia’s notorious history of extinctions and declines in small to medium sized native mammals^6^, yet this is the first study to directly compare the odour profiles of Australian marsupial and placental carnivores. Numerous experimental tests of prey naivety (e.g.^12–15,59^) have shown that native prey fail to recognise alien predator cues, which our results suggest may be due to alien predators emitting novel cues.

The finding that urine, scats, and bedding odours differ significantly between marsupial and placental carnivores also supports the archetype hypothesis of Cox and Lima^19^. This hypothesis suggests that prey will be naive to novel predator archetypes, which the authors define as a set of predators that use similar means of hunting for and capturing prey^19^. Similarities among predators grouped at the taxonomic level of Family are proposed as a practical proxy for an archetype^19^, and our results suggest that differences among predator archetypes extend to odour profiles. Despite both being terrestrial mammalian carnivores, marsupials and placentals (separated on the evolutionary tree by approximately 160 million years^28^) produce distinctly different volatile chemicals in their olfactory products (Fig. 1). Thus, prey naivety may not only be driven by differences in behaviour or hunting mode of a novel predator archetype, but also by novelty of the olfactory information that is available for prey. Together, the two hypotheses of predator archetypes^19^ and cue novelty^4,24^ suggest a probable mechanism for naivety in Australian prey: an evolutionary history with marsupial carnivores has not equipped prey with the necessary perceptual and cognitive capabilities to discriminate placental carnivore cues.

Predator urine, scats and body odour may contain a range of information in the form of intentional cues for conspecifics, and/or unintentional cues eavesdropped on by prey to enhance antipredator decision-making^22,32,45,47,51,60^. Compounds in olfactory products acting as intentional signals are likely to differ among species and among higher taxonomic levels, because differentiation of socially informative cues (e.g. those used to recognise kin, direct mating behaviour, and so on^32^); is one of the mechanisms through which species maintain reproductive isolation^36^. The accurate identification of individual potential chemical signals among the ~900 compounds found in our samples was not feasible for this study^45,47^. However, the differences in the chemical profiles of individual predator species that we report here suggest that each predator species has their own “signature mixture”^32^ of volatile compounds. Prey eavesdrop on predator cues, meaning that predator chemical signals are adapted to maximise detectability by conspecifics, not by prey^32^. It is therefore unlikely that prey use individual signalling compounds to recognise their predators, but rather that prey would associate a predator species’ signature mixture of volatile compounds (which we have analysed here as a chemical profile) with previous experiences of danger, over either ontogeny (via learning) or evolutionary time (innate recognition through adaptation)^32,60,61^. There are inevitable differences between what our sampling methodology can detect and what a given prey species would be able to detect, although we chose SPME GC-MS because it is the most sensitive and accurate analysis method currently available, and the most analogous to mammalian olfaction^62^. At the same time, mammalian predators are most likely to “shout rather than whisper” their chemical messages to conspecifics – meaning that predator chemical signals are most likely well within the range of our analytical methods^45,47,50^, and that the species- and taxon-specific signature mixes observed here are expected to be ecologically and evolutionarily relevant to prey. These species-specific signature mixtures also suggest that there is a role for olfactory cues as biologically meaningful ways to differentiate species – much as has been done for visual appearance and vocalisations.

If we consider novel alien predators to be a kind of “evolutionary trap”^63,64^, then animals are more likely to get trapped – i.e., ignore or fail to avoid the predator – if the predator is very novel, and/or the prey animal has a high response threshold for predators. Prey are less likely to recognise an alien predator’s cue if it is very different to native predator cues, and especially if predator cues have been very reliably discriminable from non-threatening cues in the prey’s evolutionary history^3,4,24^. We cannot say how reliable predator cues were for Australian native prey in the evolutionary past, although experimental evidence shows native prey do recognise and respond to native predator odours^65,66^. Our results do, however, show that the chemical profiles of placental urine, scats and bedding are fundamentally different from those of marsupials (Fig. 1), and that therefore, a mismatch between the capabilities of native prey and the cues emitted by novel placental predators is a highly plausible mechanistic explanation for native prey naivety in this system.

Invasive alien predators are a global problem, and mismatches between cues and prey detection systems likely play a similar role in other systems worldwide. Other studies have found increasing differences in chemical cues with increasing phylogenetic distance, for felids and lemurs^37,38^. At the same time, there is evidence that increasing invader impacts are correlated with increasing phylogenetic distinctiveness in grasses and in aquatic ecosystems^67,68^. If these two lines of evidence represent broad-scale general patterns across taxa and ecosystems, then naivety underpinned by cue novelty based on phylogenetic distinctiveness from the recipient community may be a mechanistic explanation for invader impacts in many environments. This idea deserves further exploration at the global scale, across ecosystem types and taxonomic groups.

The only predators with similar chemical profiles across all three odour types were dingoes and domestic dogs. Dingoes and domestic dogs are so closely related that they readily interbreed in the wild^69^. Domestic dogs and dingoes from sanctuaries consumed different diets in our study (see details in Supplementary Tables S2–S4, Methods), indicating that diet does not explain the overlap in dingo and dog chemical profiles. If dingo and dog scats, urine and body odours are chemically similar, it may facilitate native prey recognition of domestic dogs as predators in Australia. Native prey may generalise their recognition of dingo odour to that of domestic dogs, as described by the “Predator Recognition Continuum” hypothesis^70^. If true, this hypothesis would predict that Australian native prey mammals are not naive to the predation risk of domestic dogs. Previous work has shown that native bush rats (*Rattus fuscipes*) and bandicoots (*Perameles nasuta* and *Isoodon macrourus*) do appear to recognise predation risk from domestic dogs but not cats, despite having approximately 150 years’ experience with each of these predators. This may be due to native prey having ~4000 years of experience with dingoes, and the finding that dingoes and dogs have similar odour profiles supports this hypothesis^58,71,72^.

It is difficult to say why devil and fox scat chemical profiles were not significantly different (Fig. 1, Table 1). The lack of overlap between devils and foxes on the nMDS plot (Fig. 1) is indicative of a difference in composition. However, the devil scat profiles in particular are spread out widely across the plot, suggesting a high level of variation in devil scat chemical profiles may explain the lack of statistical difference between devil and fox scat chemical profiles.

Finally, while our study has focused on the implications of differing odour cues for recognition of alien predators by native prey, it is also possible for alien prey species to be naïve towards native predators (e.g.^73^), and for such naivety to result in biotic resistance to invasions by alien prey species^74,75^. The probability of alien prey being naïve towards native predators will be higher where native predators produce novel cues by comparison with familiar predators from the prey species’ native range^11^. Thus, we might expect that in Australia, placental alien prey species such as rabbits (*Oryctolagus cuniculus*) and rats (black rats - *Rattus rattus* and/or brown rats - *Rattus norvegicus*) will not recognise the olfactory cues of Australian native marsupial predators such as quolls. Barrio *et al*.^73^ confirmed this prediction for rabbits in Australia, showing that they responded to odours from evolutionarily familiar introduced foxes but not evolutionarily unfamiliar native quolls. Carthey^76^ tested black rats for responses to a range of native and non-native predator olfactory cues in Australia, finding that exotic black rats sniffed (evolutionarily familiar) exotic predator odours more than (evolutionarily unfamiliar) native predator odours. To our knowledge, no one has yet tested these ideas with invasive brown rats. It is possible that exotic prey species encountering highly novel native predator cues in the invaded environment may be naïve towards native predators, and so suffer heavy predation that acts as biotic resistance^75^. However, while some studies have shown that native predators consume and even prefer exotic over native prey species (e.g.^77,78^ but see^79^), to our knowledge native predator preference for exotic prey has not yet been demonstrably linked to the exotic prey’s naivety.

Our findings may be particularly important in understanding social communication within and among macrosmatic species. The odour profiles of placental predators are fundamentally different to those of native marsupial predators. If phylogenetically distinct alien predators invade a native ecosystem, there is a good chance that they will produce novel cues, that native prey will fail to discriminate them, and that this mechanism contributes greatly to the devastating impacts of alien predators in invaded ecosystems worldwide.

## Electronic supplementary material

Supplementary material_Tables S1-S4

Supplementary material_Data 1
