# Supplementary material for: Novel predators emit novel cues: a mechanism for prey naivety towards alien predators
Source: Sci Rep. 2017 Nov 27;7:16377. doi: 10.1038/s41598-017-16656-z (PMC5703908; doi:10.1038/s41598-017-16656-z)
Supplement: Supplementary file 1 — Supplementary material_Tables S1-S4 [file 41598_2017_16656_MOESM1_ESM.pdf]

Novel predators emit novel cues: a mechanism for prey naivety towards alien predators

Alexandra J. R. Carthey[1]*, Martin P. Bucknall[2], Kaja Wierucka[1,3], Peter B. Banks[4]

[1] Department of Biological Sciences, Macquarie University, Sydney, Australia, 2109.

[2] Bioanalytical Mass Spectrometry Facility, Mark Wainwright Analytical Centre, The University of New South Wales, Sydney, Australia, 2052.

[3] Institut des Neurosciences Paris-Saclay, Université Paris-Saclay, CNRS (UMR 9197), Université Paris-Sud, Orsay, France, 91405.

[4] School of Life and Environmental Sciences, The University of Sydney, Sydney, Australia, 2006.

* Corresponding author: Alexandra J.R. Carthey. (a): Department of Biological Sciences, Macquarie University, Sydney, NSW, 2109. (+612) 9850 9180. alexandra.carthey@mq.edu.au

Supplementary material

Table S1. Percentage contributions of each compound to the observed differences in chemical profiles of marsupial and placental predator urine, scat and bedding samples, according to SIMPER [percentage similarity analysis, conducted in PRIMER v6; 53]. Table notations: No. – numbered according to SIMPER results (contributions to differences); C – compound (numbered in order of increasing retention times); Contr – contribution of a given compound to the observed differences; CS – cumulative contribution.

	URINE				SCATS				BEDDING			
**No.**	**C**	**Contr**	**SD**	**CS**	**C**	**Contr**	**SD**	**CS**	**C**	**Contr**	**SD**	**CS**
**1**	C188	0.023	0.02	0.026	C82	0.01	0.006	0.017	C249	0.023	0.023	0.026
**2**	C32	0.016	0.012	0.043	C297	0.009	0.008	0.031	C51	0.017	0.016	0.046
**3**	C6	0.015	0.012	0.059	C56	0.006	0.006	0.042	C2	0.016	0.026	0.064
**4**	C91	0.014	0.012	0.075	C4	0.006	0.004	0.051	C8	0.016	0.022	0.081
**5**	C48	0.014	0.01	0.09	C264	0.005	0.004	0.06	C218	0.015	0.02	0.098
**6**	C17	0.013	0.016	0.105	C84	0.005	0.003	0.069	C106	0.014	0.016	0.114
**7**	C26	0.013	0.009	0.119	C32	0.005	0.005	0.077	C181	0.014	0.015	0.13
**8**	C16	0.013	0.01	0.133	C201	0.005	0.005	0.086	C5	0.014	0.015	0.146
**9**	C34	0.012	0.009	0.146	C9	0.005	0.005	0.094	C129	0.014	0.017	0.161
**10**	C30	0.011	0.014	0.159	C97	0.005	0.003	0.102	C156	0.014	0.016	0.176
**11**	C47	0.011	0.009	0.171	C22	0.005	0.003	0.11	C15	0.013	0.018	0.191
**12**	C323	0.011	0.01	0.183	C44	0.005	0.004	0.118	C6	0.013	0.015	0.206
**13**	C253	0.01	0.01	0.194	C66	0.005	0.004	0.126	C35	0.013	0.015	0.22
**14**	C75	0.009	0.01	0.204	C265	0.005	0.004	0.134	C192	0.012	0.015	0.234
**15**	C5	0.009	0.007	0.214	C13	0.005	0.003	0.142	C153	0.012	0.018	0.247
**16**	C49	0.009	0.01	0.224	C47	0.005	0.003	0.15	C1	0.012	0.015	0.261
**17**	C65	0.009	0.009	0.234	C46	0.005	0.004	0.157	C260	0.011	0.021	0.274
**18**	C80	0.009	0.008	0.244	C83	0.005	0.003	0.165	C123	0.011	0.019	0.286
**19**	C50	0.009	0.008	0.253	C65	0.005	0.004	0.173	C37	0.011	0.013	0.298
**20**	C28	0.008	0.008	0.263	C41	0.005	0.003	0.18	C73	0.01	0.012	0.31
**21**	C51	0.008	0.009	0.272	C59	0.005	0.007	0.187	C23	0.01	0.013	0.321
**22**	C9	0.008	0.006	0.281	C109	0.005	0.003	0.195	C222	0.01	0.017	0.332
**23**	C237	0.008	0.009	0.29	C48	0.004	0.003	0.202	C69	0.009	0.014	0.342

	URINE				SCATS				BEDDING			
**No.**	**C**	**Contr**	**SD**	**CS**	**C**	**Contr**	**SD**	**CS**	**C**	**Contr**	**SD**	**CS**
**24**	C7	0.008	0.012	0.298	C63	0.004	0.004	0.21	C30	0.009	0.014	0.352
**25**	C31	0.008	0.009	0.307	C62	0.004	0.005	0.217	C117	0.009	0.01	0.362
**26**	C232	0.008	0.006	0.316	C67	0.004	0.004	0.224	C4	0.009	0.016	0.372
**27**	C15	0.008	0.007	0.324	C258	0.004	0.003	0.231	C134	0.009	0.016	0.382
**28**	C25	0.008	0.012	0.333	C38	0.004	0.003	0.238	C39	0.008	0.015	0.391
**29**	C90	0.008	0.008	0.341	C69	0.004	0.004	0.245	C148	0.008	0.014	0.4
**30**	C27	0.007	0.011	0.349	C144	0.004	0.004	0.252	C139	0.008	0.017	0.409
**31**	C44	0.007	0.006	0.357	C86	0.004	0.004	0.259	C71	0.008	0.017	0.418
**32**	C186	0.007	0.012	0.364	C28	0.004	0.004	0.266	C128	0.008	0.009	0.427
**33**	C159	0.007	0.009	0.372	C85	0.004	0.003	0.273	C112	0.008	0.009	0.436
**34**	C131	0.007	0.007	0.38	C70	0.004	0.005	0.28	C49	0.007	0.011	0.444
**35**	C20	0.007	0.007	0.387	C91	0.004	0.003	0.286	C199	0.007	0.016	0.452
**36**	C142	0.007	0.006	0.395	C10	0.004	0.003	0.293	C25	0.007	0.011	0.46
**37**	C100	0.007	0.01	0.402	C112	0.004	0.003	0.299	C41	0.007	0.011	0.468
**38**	C197	0.006	0.007	0.409	C11	0.004	0.003	0.306	C223	0.007	0.01	0.476
**39**	C295	0.006	0.007	0.416	C276	0.004	0.003	0.312	C188	0.007	0.019	0.483
**40**	C14	0.006	0.007	0.424	C99	0.004	0.003	0.319	C247	0.006	0.009	0.49
**41**	C179	0.006	0.008	0.431	C104	0.004	0.003	0.325	C158	0.006	0.013	0.497
**42**	C105	0.006	0.006	0.438	C108	0.004	0.002	0.331	C159	0.006	0.014	0.504
**43**	C151	0.006	0.005	0.444	C132	0.004	0.003	0.337	C164	0.006	0.009	0.51
**44**	C128	0.006	0.006	0.451	C35	0.004	0.003	0.344	C230	0.006	0.01	0.517
**45**	C254	0.006	0.005	0.458	C25	0.004	0.003	0.35	C166	0.006	0.01	0.523
**46**	C306	0.006	0.007	0.465	C52	0.004	0.004	0.356	C133	0.005	0.011	0.529
**47**	C216	0.006	0.01	0.471	C162	0.004	0.003	0.362	C67	0.005	0.016	0.535
**48**	C52	0.006	0.006	0.478	C90	0.004	0.004	0.368	C12	0.005	0.011	0.541
**49**	C77	0.006	0.008	0.484	C80	0.004	0.004	0.374	C233	0.005	0.013	0.547
**50**	C120	0.006	0.008	0.491	C23	0.004	0.004	0.38	C137	0.005	0.013	0.553
**51**	C221	0.006	0.005	0.497	C21	0.004	0.003	0.386	C227	0.005	0.008	0.559
**52**	C103	0.006	0.007	0.503	C19	0.004	0.003	0.392	C16	0.005	0.018	0.565
**53**	C18	0.005	0.008	0.509	C160	0.004	0.003	0.398	C54	0.005	0.01	0.57
**54**	C13	0.005	0.01	0.515	C49	0.004	0.002	0.404	C235	0.005	0.008	0.576
**55**	C29	0.005	0.01	0.521	C179	0.004	0.004	0.409	C27	0.005	0.014	0.581
**56**	C229	0.005	0.006	0.526	C96	0.003	0.003	0.415	C225	0.005	0.011	0.587

	URINE				SCATS				BEDDING			
**No.**	**C**	**Contr**	**SD**	**CS**	**C**	**Contr**	**SD**	**CS**	**C**	**Contr**	**SD**	**CS**
**57**	C66	0.005	0.006	0.532	C143	0.003	0.003	0.421	C205	0.005	0.017	0.592
**58**	C35	0.005	0.006	0.538	C210	0.003	0.004	0.427	C170	0.005	0.011	0.598
**59**	C274	0.005	0.007	0.543	C221	0.003	0.003	0.432	C228	0.005	0.007	0.603
**60**	C85	0.005	0.006	0.549	C174	0.003	0.003	0.438	C61	0.005	0.01	0.608
**61**	C129	0.005	0.006	0.554	C195	0.003	0.002	0.443	C60	0.005	0.008	0.614
**62**	C228	0.005	0.006	0.559	C40	0.003	0.003	0.449	C217	0.005	0.008	0.619
**63**	C168	0.005	0.006	0.564	C281	0.003	0.003	0.454	C176	0.005	0.01	0.624
**64**	C256	0.005	0.009	0.569	C110	0.003	0.002	0.459	C14	0.005	0.007	0.629
**65**	C147	0.005	0.007	0.575	C74	0.003	0.004	0.465	C38	0.005	0.008	0.634
**66**	C72	0.005	0.006	0.58	C12	0.003	0.003	0.47	C160	0.004	0.009	0.639
**67**	C73	0.004	0.008	0.585	C42	0.003	0.003	0.475	C13	0.004	0.009	0.644
**68**	C121	0.004	0.005	0.589	C37	0.003	0.003	0.48	C241	0.004	0.006	0.649
**69**	C257	0.004	0.009	0.594	C103	0.003	0.002	0.486	C167	0.004	0.007	0.654
**70**	C154	0.004	0.005	0.599	C5	0.003	0.003	0.491	C242	0.004	0.011	0.658
**71**	C214	0.004	0.006	0.603	C105	0.003	0.002	0.496	C236	0.004	0.011	0.663
**72**	C175	0.004	0.008	0.608	C242	0.003	0.004	0.501	C108	0.004	0.007	0.668
**73**	C115	0.004	0.005	0.612	C182	0.003	0.003	0.506	C58	0.004	0.009	0.672
**74**	C108	0.004	0.006	0.617	C6	0.003	0.003	0.511	C81	0.004	0.012	0.677
**75**	C97	0.004	0.007	0.621	C16	0.003	0.003	0.516	C219	0.004	0.006	0.681
**76**	C157	0.004	0.005	0.626	C98	0.003	0.003	0.521	C212	0.004	0.006	0.686
**77**	C55	0.004	0.007	0.63	C129	0.003	0.002	0.526	C224	0.004	0.006	0.69
**78**	C202	0.004	0.005	0.634	C7	0.003	0.003	0.531	C195	0.004	0.009	0.695
**79**	C283	0.004	0.005	0.639	C60	0.003	0.002	0.536	C210	0.004	0.006	0.699
**80**	C294	0.004	0.008	0.643	C170	0.003	0.003	0.54	C252	0.004	0.006	0.703
**81**	C134	0.004	0.005	0.647	C125	0.003	0.002	0.545	C96	0.004	0.006	0.707
**82**	C322	0.004	0.006	0.651	C203	0.003	0.002	0.55	C246	0.003	0.006	0.711
**83**	C174	0.004	0.005	0.655	C45	0.003	0.002	0.555	C198	0.003	0.006	0.715
**84**	C286	0.004	0.006	0.66	C199	0.003	0.003	0.559	C55	0.003	0.012	0.719
**85**	C234	0.004	0.007	0.664	C18	0.003	0.002	0.564	C46	0.003	0.008	0.723
**86**	C19	0.004	0.005	0.668	C252	0.003	0.002	0.569	C114	0.003	0.009	0.727
**87**	C242	0.004	0.006	0.672	C194	0.003	0.003	0.573	C221	0.003	0.007	0.731
**88**	C199	0.004	0.005	0.676	C164	0.003	0.003	0.578	C87	0.003	0.008	0.734
**89**	C81	0.004	0.006	0.68	C1	0.003	0.002	0.582	C237	0.003	0.006	0.738

	URINE				SCATS				BEDDING			
**No.**	**C**	**Contr**	**SD**	**CS**	**C**	**Contr**	**SD**	**CS**	**C**	**Contr**	**SD**	**CS**
**90**	C236	0.004	0.005	0.684	C17	0.003	0.002	0.587	C254	0.003	0.006	0.742
**91**	C102	0.003	0.005	0.688	C172	0.003	0.003	0.591	C207	0.003	0.006	0.746
**92**	C231	0.003	0.008	0.691	C298	0.003	0.004	0.596	C22	0.003	0.012	0.749
**93**	C176	0.003	0.006	0.695	C299	0.003	0.002	0.6	C208	0.003	0.006	0.753
**94**	C171	0.003	0.005	0.699	C222	0.003	0.004	0.605	C216	0.003	0.005	0.757
**95**	C124	0.003	0.005	0.703	C227	0.003	0.003	0.609	C244	0.003	0.005	0.76
**96**	C98	0.003	0.005	0.706	C236	0.003	0.002	0.614	C264	0.003	0.005	0.764
**97**	C271	0.003	0.006	0.71	C78	0.003	0.003	0.618	C213	0.003	0.007	0.767
**98**	C185	0.003	0.004	0.714	C192	0.003	0.003	0.622	C20	0.003	0.007	0.77
**99**	C111	0.003	0.005	0.717	C138	0.003	0.002	0.627	C259	0.003	0.005	0.774
**100**	C273	0.003	0.004	0.721	C142	0.003	0.002	0.631	C256	0.003	0.005	0.777
**101**	C282	0.003	0.006	0.724	C290	0.003	0.002	0.635	C66	0.003	0.004	0.78
**102**	C165	0.003	0.006	0.728	C188	0.003	0.002	0.64	C258	0.003	0.005	0.783
**103**	C298	0.003	0.004	0.731	C8	0.003	0.002	0.644	C151	0.003	0.01	0.786
**104**	C153	0.003	0.004	0.735	C3	0.003	0.002	0.648	C3	0.003	0.006	0.789
**105**	C163	0.003	0.005	0.738	C87	0.002	0.004	0.652	C135	0.003	0.007	0.792
**106**	C125	0.003	0.006	0.741	C75	0.002	0.002	0.656	C163	0.003	0.005	0.795
**107**	C57	0.003	0.005	0.744	C247	0.002	0.002	0.66	C250	0.003	0.005	0.798
**108**	C11	0.003	0.005	0.747	C94	0.002	0.002	0.664	C140	0.003	0.006	0.801
**109**	C45	0.003	0.004	0.75	C122	0.002	0.002	0.669	C257	0.003	0.004	0.803
**110**	C191	0.003	0.006	0.753	C131	0.002	0.002	0.673	C116	0.002	0.009	0.806
**111**	C219	0.003	0.005	0.756	C207	0.002	0.002	0.677	C202	0.002	0.007	0.809
**112**	C284	0.003	0.005	0.759	C20	0.002	0.002	0.681	C234	0.002	0.004	0.812
**113**	C220	0.003	0.005	0.761	C263	0.002	0.002	0.685	C168	0.002	0.005	0.814
**114**	C84	0.003	0.006	0.764	C197	0.002	0.003	0.688	C77	0.002	0.006	0.817
**115**	C83	0.003	0.005	0.767	C39	0.002	0.002	0.692	C209	0.002	0.005	0.82
**116**	C210	0.003	0.004	0.77	C158	0.002	0.002	0.696	C267	0.002	0.005	0.822
**117**	C314	0.003	0.004	0.773	C230	0.002	0.002	0.7	C44	0.002	0.008	0.825
**118**	C269	0.003	0.006	0.776	C249	0.002	0.002	0.704	C174	0.002	0.011	0.827
**119**	C268	0.003	0.004	0.778	C14	0.002	0.003	0.708	C74	0.002	0.006	0.83
**120**	C258	0.003	0.005	0.781	C270	0.002	0.002	0.711	C273	0.002	0.005	0.833
**121**	C93	0.002	0.005	0.784	C274	0.002	0.003	0.715	C262	0.002	0.005	0.835
**122**	C63	0.002	0.004	0.787	C279	0.002	0.002	0.719	C266	0.002	0.005	0.838

	URINE				SCATS				BEDDING			
**No.**	**C**	**Contr**	**SD**	**CS**	**C**	**Contr**	**SD**	**CS**	**C**	**Contr**	**SD**	**CS**
**123**	C208	0.002	0.005	0.789	C120	0.002	0.002	0.722	C80	0.002	0.007	0.84
**124**	C244	0.002	0.005	0.792	C128	0.002	0.002	0.726	C63	0.002	0.007	0.842
**125**	C205	0.002	0.006	0.794	C212	0.002	0.002	0.729	C184	0.002	0.004	0.845
**126**	C200	0.002	0.005	0.796	C117	0.002	0.002	0.733	C78	0.002	0.01	0.847
**127**	C64	0.002	0.004	0.799	C178	0.002	0.004	0.737	C53	0.002	0.007	0.849
**128**	C252	0.002	0.005	0.801	C153	0.002	0.002	0.74	C115	0.002	0.007	0.852
**129**	C173	0.002	0.004	0.803	C137	0.002	0.002	0.744	C84	0.002	0.006	0.854
**130**	C99	0.002	0.009	0.806	C2	0.002	0.002	0.747	C243	0.002	0.01	0.856
**131**	C267	0.002	0.004	0.808	C106	0.002	0.002	0.75	C76	0.002	0.007	0.858
**132**	C259	0.002	0.006	0.81	C15	0.002	0.002	0.754	C107	0.002	0.007	0.86
**133**	C114	0.002	0.005	0.813	C50	0.002	0.002	0.757	C24	0.002	0.006	0.863
**134**	C76	0.002	0.005	0.815	C72	0.002	0.002	0.761	C265	0.002	0.004	0.865
**135**	C110	0.002	0.004	0.817	C141	0.002	0.002	0.764	C215	0.002	0.004	0.867
**136**	C233	0.002	0.004	0.819	C53	0.002	0.002	0.767	C68	0.002	0.006	0.869
**137**	C22	0.002	0.004	0.821	C171	0.002	0.002	0.77	C162	0.002	0.004	0.871
**138**	C264	0.002	0.004	0.823	C168	0.002	0.002	0.774	C173	0.002	0.004	0.873
**139**	C106	0.002	0.004	0.826	C235	0.002	0.002	0.777	C65	0.002	0.006	0.874
**140**	C169	0.002	0.003	0.828	C113	0.002	0.002	0.78	C105	0.002	0.003	0.876
**141**	C127	0.002	0.003	0.83	C217	0.002	0.003	0.783	C180	0.002	0.006	0.878
**142**	C130	0.002	0.004	0.832	C273	0.002	0.002	0.787	C130	0.002	0.005	0.88
**143**	C43	0.002	0.005	0.834	C205	0.002	0.004	0.79	C251	0.002	0.003	0.882
**144**	C299	0.002	0.005	0.836	C140	0.002	0.002	0.793	C11	0.002	0.008	0.884
**145**	C241	0.002	0.006	0.838	C95	0.002	0.002	0.796	C57	0.002	0.005	0.885
**146**	C247	0.002	0.004	0.84	C198	0.002	0.003	0.799	C191	0.002	0.003	0.887
**147**	C279	0.002	0.004	0.841	C92	0.002	0.003	0.802	C85	0.002	0.005	0.889
**148**	C278	0.002	0.005	0.843	C155	0.002	0.002	0.805	C177	0.002	0.007	0.891
**149**	C82	0.002	0.005	0.845	C229	0.002	0.003	0.808	C113	0.002	0.006	0.892
**150**	C318	0.002	0.003	0.847	C163	0.002	0.001	0.811	C86	0.002	0.005	0.894
**151**	C193	0.002	0.005	0.849	C266	0.002	0.002	0.814	C33	0.002	0.005	0.896
**152**	C262	0.002	0.004	0.851	C76	0.002	0.002	0.817	C7	0.001	0.007	0.897
**153**	C12	0.002	0.005	0.853	C118	0.002	0.003	0.82	C127	0.001	0.005	0.899
**154**	C320	0.002	0.003	0.855	C148	0.002	0.002	0.823	C141	0.001	0.007	0.901
**155**	C38	0.002	0.003	0.856	C89	0.002	0.002	0.826	C238	0.001	0.007	0.902

	URINE				SCATS				BEDDING			
**No.**	**C**	**Contr**	**SD**	**CS**	**C**	**Contr**	**SD**	**CS**	**C**	**Contr**	**SD**	**CS**
**156**	C132	0.002	0.004	0.858	C124	0.002	0.002	0.829	C48	0.001	0.007	0.904
**157**	C79	0.002	0.006	0.86	C237	0.002	0.002	0.831	C21	0.001	0.007	0.906
**158**	C101	0.002	0.003	0.862	C31	0.002	0.002	0.834	C270	0.001	0.007	0.907
**159**	C36	0.002	0.005	0.864	C100	0.002	0.003	0.837	C82	0.001	0.007	0.909
**160**	C213	0.002	0.004	0.865	C146	0.002	0.002	0.839	C34	0.001	0.005	0.91
**161**	C40	0.002	0.005	0.867	C219	0.002	0.002	0.842	C240	0.001	0.007	0.912
**162**	C170	0.002	0.005	0.869	C169	0.002	0.002	0.845	C155	0.001	0.003	0.913
**163**	C96	0.002	0.007	0.87	C213	0.002	0.002	0.847	C59	0.001	0.004	0.915
**164**	C250	0.002	0.003	0.872	C262	0.002	0.002	0.85	C179	0.001	0.003	0.916
**165**	C3	0.002	0.004	0.874	C193	0.002	0.002	0.853	C52	0.001	0.004	0.918
**166**	C296	0.002	0.004	0.876	C204	0.002	0.003	0.855	C271	0.001	0.003	0.919
**167**	C119	0.001	0.004	0.877	C134	0.002	0.002	0.858	C239	0.001	0.003	0.921
**168**	C146	0.001	0.004	0.879	C93	0.002	0.003	0.86	C97	0.001	0.004	0.922
**169**	C189	0.001	0.003	0.88	C231	0.002	0.002	0.863	C268	0.001	0.003	0.923
**170**	C195	0.001	0.003	0.882	C244	0.002	0.002	0.865	C146	0.001	0.003	0.925
**171**	C148	0.001	0.006	0.884	C189	0.002	0.002	0.868	C119	0.001	0.004	0.926
**172**	C158	0.001	0.005	0.885	C238	0.001	0.002	0.87	C149	0.001	0.003	0.928
**173**	C161	0.001	0.004	0.887	C246	0.001	0.002	0.873	C157	0.001	0.003	0.929
**174**	C206	0.001	0.006	0.888	C215	0.001	0.002	0.875	C226	0.001	0.006	0.93
**175**	C152	0.001	0.004	0.89	C239	0.001	0.002	0.877	C150	0.001	0.003	0.932
**176**	C140	0.001	0.006	0.891	C216	0.001	0.002	0.879	C143	0.001	0.003	0.933
**177**	C222	0.001	0.004	0.893	C224	0.001	0.002	0.882	C142	0.001	0.006	0.934
**178**	C113	0.001	0.003	0.894	C156	0.001	0.002	0.884	C194	0.001	0.003	0.935
**179**	C277	0.001	0.004	0.896	C211	0.001	0.003	0.886	C182	0.001	0.003	0.937
**180**	C150	0.001	0.006	0.897	C107	0.001	0.002	0.888	C101	0.001	0.003	0.938
**181**	C281	0.001	0.003	0.899	C149	0.001	0.002	0.89	C175	0.001	0.003	0.939
**182**	C54	0.001	0.006	0.9	C123	0.001	0.002	0.892	C161	0.001	0.005	0.941
**183**	C58	0.001	0.004	0.902	C283	0.001	0.002	0.894	C186	0.001	0.006	0.942
**184**	C2	0.001	0.004	0.903	C151	0.001	0.002	0.896	C193	0.001	0.005	0.943
**185**	C144	0.001	0.003	0.904	C190	0.001	0.002	0.898	C255	0.001	0.005	0.944
**186**	C251	0.001	0.004	0.906	C186	0.001	0.002	0.9	C245	0.001	0.005	0.945
**187**	C37	0.001	0.003	0.907	C61	0.001	0.002	0.902	C187	0.001	0.003	0.947
**188**	C308	0.001	0.003	0.908	C24	0.001	0.002	0.904	C229	0.001	0.005	0.948

	URINE				SCATS				BEDDING			
**No.**	**C**	**Contr**	**SD**	**CS**	**C**	**Contr**	**SD**	**CS**	**C**	**Contr**	**SD**	**CS**
**189**	C302	0.001	0.003	0.91	C154	0.001	0.002	0.906	C248	0.001	0.003	0.949
**190**	C190	0.001	0.003	0.911	C253	0.001	0.002	0.908	C211	0.001	0.005	0.95
**191**	C223	0.001	0.005	0.912	C202	0.001	0.002	0.909	C98	0.001	0.003	0.951
**192**	C239	0.001	0.004	0.914	C214	0.001	0.002	0.911	C232	0.001	0.005	0.952
**193**	C297	0.001	0.003	0.915	C64	0.001	0.003	0.913	C126	0.001	0.003	0.954
**194**	C8	0.001	0.005	0.916	C275	0.001	0.002	0.914	C19	0.001	0.005	0.955
**195**	C164	0.001	0.005	0.917	C187	0.001	0.002	0.916	C10	0.001	0.004	0.956
**196**	C24	0.001	0.003	0.919	C271	0.001	0.002	0.918	C62	0.001	0.004	0.957
**197**	C94	0.001	0.004	0.92	C55	0.001	0.002	0.919	C231	0.001	0.004	0.958
**198**	C280	0.001	0.005	0.921	C183	0.001	0.002	0.921	C102	0.001	0.004	0.959
**199**	C69	0.001	0.003	0.922	C167	0.001	0.002	0.923	C201	0.001	0.004	0.96
**200**	C248	0.001	0.003	0.923	C121	0.001	0.002	0.924	C136	0.001	0.004	0.961
**201**	C263	0.001	0.002	0.925	C58	0.001	0.003	0.926	C75	0.001	0.004	0.962
**202**	C301	0.001	0.002	0.926	C57	0.001	0.002	0.928	C152	0.001	0.004	0.963
**203**	C184	0.001	0.003	0.927	C206	0.001	0.002	0.929	C185	0.001	0.004	0.964
**204**	C126	0.001	0.004	0.928	C26	0.001	0.002	0.931	C70	0.001	0.003	0.965
**205**	C246	0.001	0.003	0.929	C101	0.001	0.002	0.932	C206	0.001	0.004	0.966
**206**	C162	0.001	0.004	0.93	C68	0.001	0.002	0.934	C269	0.001	0.004	0.966
**207**	C226	0.001	0.004	0.931	C29	0.001	0.003	0.935	C32	0.001	0.004	0.967
**208**	C309	0.001	0.004	0.932	C185	0.001	0.002	0.937	C99	0.001	0.004	0.968
**209**	C276	0.001	0.004	0.933	C220	0.001	0.002	0.938	C132	0.001	0.004	0.969
**210**	C203	0.001	0.004	0.934	C79	0.001	0.002	0.94	C165	0.001	0.004	0.97
**211**	C53	0.001	0.004	0.935	C157	0.001	0.001	0.941	C109	0.001	0.003	0.971
**212**	C123	0.001	0.004	0.936	C51	0.001	0.002	0.942	C144	0.001	0.003	0.972
**213**	C182	0.001	0.003	0.937	C102	0.001	0.002	0.944	C94	0.001	0.003	0.972
**214**	C70	0.001	0.004	0.938	C218	0.001	0.002	0.945	C31	0.001	0.003	0.973
**215**	C290	0.001	0.003	0.939	C119	0.001	0.002	0.946	C17	0.001	0.003	0.974
**216**	C240	0.001	0.003	0.94	C71	0.001	0.002	0.948	C72	0.001	0.003	0.975
**217**	C312	0.001	0.002	0.941	C130	0.001	0.002	0.949	C120	0.001	0.003	0.975
**218**	C68	0.001	0.003	0.942	C196	0.001	0.002	0.95	C124	0.001	0.003	0.976
**219**	C303	0.001	0.003	0.943	C234	0.001	0.002	0.951	C183	0.001	0.003	0.977
**220**	C178	0.001	0.004	0.944	C223	0.001	0.002	0.953	C100	0.001	0.003	0.978
**221**	C67	0.001	0.003	0.945	C181	0.001	0.002	0.954	C89	0.001	0.003	0.979

	URINE				SCATS				BEDDING			
**No.**	**C**	**Contr**	**SD**	**CS**	**C**	**Contr**	**SD**	**CS**	**C**	**Contr**	**SD**	**CS**
**222**	C33	0.001	0.004	0.946	C295	0.001	0.003	0.955	C131	0.001	0.003	0.979
**223**	C212	0.001	0.003	0.947	C173	0.001	0.003	0.956	C197	0.001	0.002	0.98
**224**	C89	0.001	0.003	0.948	C115	0.001	0.001	0.957	C36	0.001	0.003	0.981
**225**	C133	0.001	0.002	0.949	C114	0.001	0.001	0.959	C90	0.001	0.002	0.981
**226**	C92	0.001	0.003	0.95	C126	0.001	0.001	0.96	C56	0.001	0.002	0.982
**227**	C310	0.001	0.002	0.951	C184	0.001	0.003	0.961	C125	0.001	0.002	0.983
**228**	C56	0.001	0.004	0.952	C260	0.001	0.001	0.962	C18	0.001	0.003	0.984
**229**	C156	0.001	0.002	0.952	C287	0.001	0.002	0.963	C122	0.001	0.002	0.984
**230**	C23	0.001	0.003	0.953	C111	0.001	0.002	0.964	C95	0.001	0.003	0.985
**231**	C137	0.001	0.003	0.954	C288	0.001	0.001	0.965	C43	0.001	0.002	0.986
**232**	C224	0.001	0.003	0.955	C200	0.001	0.002	0.966	C83	0.001	0.003	0.986
**233**	C41	0.001	0.003	0.956	C257	0.001	0.002	0.967	C47	0.001	0.003	0.987
**234**	C122	0.001	0.004	0.957	C284	0.001	0.001	0.968	C91	0.001	0.003	0.988
**235**	C187	0.001	0.002	0.957	C33	0.001	0.002	0.969	C200	0.001	0.002	0.988
**236**	C293	0.001	0.003	0.958	C208	0.001	0.002	0.97	C50	0.001	0.003	0.989
**237**	C207	0.001	0.003	0.959	C30	0.001	0.002	0.971	C64	0.001	0.003	0.99
**238**	C74	0.001	0.003	0.96	C116	0.001	0.001	0.972	C204	0.001	0.002	0.99
**239**	C270	0.001	0.003	0.96	C225	0.001	0.001	0.973	C29	0.001	0.002	0.991
**240**	C109	0.001	0.002	0.961	C88	0.001	0.001	0.973	C42	0.001	0.003	0.992
**241**	C71	0.001	0.002	0.962	C135	0	0.001	0.974	C121	0.001	0.002	0.992
**242**	C116	0.001	0.003	0.963	C145	0	0.002	0.975	C263	0.001	0.002	0.993
**243**	C104	0.001	0.003	0.964	C176	0	0.002	0.976	C272	0.001	0.002	0.994
**244**	C155	0.001	0.003	0.964	C34	0	0.002	0.976	C110	0.001	0.002	0.994
**245**	C60	0.001	0.002	0.965	C36	0	0.002	0.977	C88	0.001	0.002	0.995
**246**	C160	0.001	0.003	0.966	C165	0	0.002	0.978	C203	0	0.002	0.995
**247**	C95	0.001	0.002	0.966	C166	0	0.001	0.979	C172	0	0.002	0.996
**248**	C107	0.001	0.002	0.967	C209	0	0.001	0.979	C92	0	0.002	0.996
**249**	C42	0.001	0.002	0.968	C27	0	0.001	0.98	C196	0	0.002	0.997
**250**	C88	0.001	0.003	0.969	C161	0	0.001	0.981	C118	0	0.002	0.998
**251**	C86	0.001	0.003	0.969	C278	0	0.001	0.982	C171	0	0.002	0.998
**252**	C204	0.001	0.002	0.97	C150	0	0.001	0.982	C190	0	0.002	0.999
**253**	C243	0.001	0.002	0.971	C241	0	0.001	0.983	C93	0	0.002	0.999
**254**	C143	0.001	0.002	0.971	C232	0	0.002	0.983	C79	0	0.001	1

	URINE				SCATS				BEDDING			
**No.**	**C**	**Contr**	**SD**	**CS**	**C**	**Contr**	**SD**	**CS**	**C**	**Contr**	**SD**	**CS**
**255**	C167	0.001	0.002	0.972	C133	0	0.002	0.984	C103	0	0.001	1
**256**	C118	0.001	0.002	0.973	C267	0	0.001	0.985	-	-	-	-
**257**	C313	0.001	0.002	0.973	C291	0	0.001	0.985	-	-	-	-
**258**	C317	0.001	0.002	0.974	C228	0	0.001	0.986	-	-	-	-
**259**	C265	0.001	0.003	0.975	C248	0	0.002	0.987	-	-	-	-
**260**	C287	0.001	0.003	0.975	C286	0	0.001	0.987	-	-	-	-
**261**	C4	0.001	0.002	0.976	C261	0	0.001	0.988	-	-	-	-
**262**	C198	0.001	0.002	0.977	C43	0	0.001	0.988	-	-	-	-
**263**	C21	0.001	0.003	0.977	C191	0	0.001	0.989	-	-	-	-
**264**	C192	0.001	0.002	0.978	C177	0	0.001	0.989	-	-	-	-
**265**	C172	0.001	0.002	0.978	C259	0	0.001	0.99	-	-	-	-
**266**	C62	0.001	0.003	0.979	C255	0	0.001	0.99	-	-	-	-
**267**	C87	0.001	0.002	0.98	C251	0	0.001	0.991	-	-	-	-
**268**	C304	0.001	0.003	0.98	C269	0	0.001	0.991	-	-	-	-
**269**	C319	0.001	0.003	0.981	C73	0	0.001	0.992	-	-	-	-
**270**	C10	0.001	0.003	0.982	C282	0	0.001	0.992	-	-	-	-
**271**	C139	0.001	0.002	0.982	C226	0	0.001	0.992	-	-	-	-
**272**	C138	0.001	0.003	0.983	C254	0	0.001	0.993	-	-	-	-
**273**	C59	0.001	0.002	0.983	C147	0	0.001	0.993	-	-	-	-
**274**	C61	0.001	0.002	0.984	C240	0	0.001	0.994	-	-	-	-
**275**	C1	0.001	0.003	0.984	C256	0	0.001	0.994	-	-	-	-
**276**	C217	0.001	0.002	0.985	C159	0	0.001	0.994	-	-	-	-
**277**	C255	0	0.003	0.985	C152	0	0.001	0.995	-	-	-	-
**278**	C39	0	0.002	0.986	C54	0	0.001	0.995	-	-	-	-
**279**	C266	0	0.002	0.986	C127	0	0.001	0.996	-	-	-	-
**280**	C307	0	0.002	0.987	C136	0	0.001	0.996	-	-	-	-
**281**	C46	0	0.002	0.987	C180	0	0.001	0.996	-	-	-	-
**282**	C194	0	0.001	0.988	C245	0	0.001	0.997	-	-	-	-
**283**	C260	0	0.002	0.988	C250	0	0.001	0.997	-	-	-	-
**284**	C196	0	0.001	0.989	C285	0	0.001	0.997	-	-	-	-
**285**	C238	0	0.002	0.989	C139	0	0.001	0.998	-	-	-	-
**286**	C211	0	0.002	0.99	C233	0	0.001	0.998	-	-	-	-
**287**	C218	0	0.002	0.99	C81	0	0.001	0.998	-	-	-	-

	URINE				SCATS				BEDDING			
**No.**	**C**	**Contr**	**SD**	**CS**	**C**	**Contr**	**SD**	**CS**	**C**	**Contr**	**SD**	**CS**
**288**	C289	0	0.002	0.99	C294	0	0.001	0.999	-	-	-	-
**289**	C227	0	0.002	0.991	C292	0	0.001	0.999	-	-	-	-
**290**	C315	0	0.001	0.991	C268	0	0.001	0.999	-	-	-	-
**291**	C245	0	0.002	0.992	C77	0	0.001	1	-	-	-	-
**292**	C291	0	0.002	0.992	C272	0	0.001	1	-	-	-	-
**293**	C183	0	0.002	0.992	C243	0	0.001	1	-	-	-	-
**294**	C177	0	0.002	0.993	-	-	-	-	-	-	-	-
**295**	C78	0	0.002	0.993	-	-	-	-	-	-	-	-
**296**	C145	0	0.002	0.994	-	-	-	-	-	-	-	-
**297**	C311	0	0.002	0.994	-	-	-	-	-	-	-	-
**298**	C316	0	0.002	0.994	-	-	-	-	-	-	-	-
**299**	C249	0	0.002	0.995	-	-	-	-	-	-	-	-
**300**	C215	0	0.001	0.995	-	-	-	-	-	-	-	-
**301**	C225	0	0.001	0.995	-	-	-	-	-	-	-	-
**302**	C141	0	0.001	0.996	-	-	-	-	-	-	-	-
**303**	C166	0	0.001	0.996	-	-	-	-	-	-	-	-
**304**	C117	0	0.001	0.996	-	-	-	-	-	-	-	-
**305**	C272	0	0.001	0.997	-	-	-	-	-	-	-	-
**306**	C149	0	0.001	0.997	-	-	-	-	-	-	-	-
**307**	C209	0	0.001	0.997	-	-	-	-	-	-	-	-
**308**	C135	0	0.001	0.998	-	-	-	-	-	-	-	-
**309**	C275	0	0.001	0.998	-	-	-	-	-	-	-	-
**310**	C288	0	0.001	0.998	-	-	-	-	-	-	-	-
**311**	C112	0	0.001	0.998	-	-	-	-	-	-	-	-
**312**	C292	0	0.001	0.999	-	-	-	-	-	-	-	-
**313**	C201	0	0.001	0.999	-	-	-	-	-	-	-	-
**314**	C235	0	0.001	0.999	-	-	-	-	-	-	-	-
**315**	C305	0	0.001	1	-	-	-	-	-	-	-	-
**316**	C285	0	0.001	1	-	-	-	-	-	-	-	-
**317**	C230	0	0.001	1	-	-	-	-	-	-	-	-

TABLE S2. Source type, animal age, sex, and de-sexing status for urine samples.

Predator	Sex	Desexing status	Age	Source type
Dingo	Female	Entire	Adult	Domestic
Dingo	Female	Entire	Adult	Domestic
Dingo	Male	Desexed	Adult	Domestic
Dingo	Male	Entire	Juvenile	Domestic
Dingo	Male	Entire	Adult	Domestic
Dingo	Male	Entire	Adult	Sanctuary/Zoo
Dingo	Male	Desexed	Adult	Sanctuary/Zoo
Dog	Female	Entire	Adult	Domestic
Dog	Female	Entire	Adult	Domestic
Dog	Female	Entire	Juvenile	Domestic
Dog	Female	Entire	Adult	Domestic
Dog	Male	Entire	Adult	Domestic
Dog	Female	Desexed	Adult	Domestic
Cat	Female	Entire	Adult	Domestic
Cat	Female	Entire	Adult	Domestic
Cat	Female	Entire	Juvenile	Domestic
Cat	Female	Entire	Juvenile	Domestic
Cat	Female	Entire	Juvenile	Domestic
Cat	Female	Entire	Juvenile	Domestic
Eastern quoll	Female	Entire	Adult	Sanctuary/Zoo
Eastern quoll	Male	Entire	Adult	Sanctuary/Zoo
Eastern quoll	Male	Entire	Adult	Sanctuary/Zoo
Tiger quoll	Female	Entire	Adult	Sanctuary/Zoo
Tiger quoll	Male	Entire	Adult	Sanctuary/Zoo
Tiger quoll	Female	Entire	Adult	Sanctuary/Zoo
Tiger quoll	Female	Entire	Adult	Sanctuary/Zoo
Tiger quoll	Male	Entire	Adult	Sanctuary/Zoo
Tiger quoll	Male	Entire	Adult	Sanctuary/Zoo
Devil	Male	Entire	Adult	Sanctuary/Zoo
Devil	Male	Entire	Adult	Sanctuary/Zoo
Devil	Male	Entire	Adult	Sanctuary/Zoo
Devil	Male	Entire	Adult	Sanctuary/Zoo
Devil	Female	Entire	Adult	Sanctuary/Zoo
Devil	Female	Entire	Adult	Sanctuary/Zoo
Devil	Male	Entire	Adult	Sanctuary/Zoo
Fox	Female	Desexed	Adult	Domestic
Fox	Female	Entire	Adult	Wild
Fox	Male	Entire	Adult	Wild
Fox	Male	Entire	Adult	Wild
Fox	Female	Entire	Adult	Wild

TABLE S3. Source type, animal age, sex, and de-sexing status for scat samples.

Predator	Sex	Desexing status	Age	Source type
Dingo	Female	Entire	Adult	Domestic
Dingo	Female	Entire	Adult	Domestic
Dingo	Male	Desexed	Adult	Domestic
Dingo	Male	Entire	Juvenile	Domestic
Dingo	Male	Entire	Adult	Domestic
Dingo	Male	Entire	Adult	Sanctuary/Zoo
Dingo	Male	Desexed	Adult	Sanctuary/Zoo
Dog	Male	Desexed	Adult	Domestic
Dog	Female	Desexed	Adult	Domestic
Dog	Male	Desexed	Adult	Domestic
Dog	Female	Desexed	Adult	Domestic
Dog	Male	Desexed	Juvenile	Domestic
Dog	Male	Desexed	Adult	Domestic
Cat	Female	Desexed	Adult	Domestic
Cat	Male	Entire	Juvenile	Domestic
Cat	Male	Entire	Juvenile	Domestic
Cat	Female	Entire	Juvenile	Domestic
Cat	Male	Entire	Juvenile	Domestic
Cat	Male	Entire	Juvenile	Domestic
Tiger quoll	Male	Entire	Adult	Sanctuary/Zoo
Tiger quoll	Male	Entire	Adult	Sanctuary/Zoo
Tiger quoll	Male	Entire	Adult	Sanctuary/Zoo
Tiger quoll	Male	Entire	Adult	Sanctuary/Zoo
Tiger quoll	Female	Entire	Adult	Sanctuary/Zoo
Tiger quoll	Female	Entire	Unknown	Sanctuary/Zoo
Devil	Male	Entire	Adult	Sanctuary/Zoo
Devil	Female	Entire	Adult	Sanctuary/Zoo
Devil	Female	Entire	Adult	Sanctuary/Zoo
Devil	Male	Entire	Adult	Sanctuary/Zoo
Devil	Female	Entire	Adult	Sanctuary/Zoo
Devil	Male	Entire	Adult	Sanctuary/Zoo
Devil	Male	Entire	Adult	Sanctuary/Zoo
Fox	Female	Desexed	Adult	Domestic
Fox	Unknown	Entire	Unknown	Wild
Fox	Female	Entire	Juvenile	Sanctuary/Zoo
Fox	Unknown	Desexed	Adult	Sanctuary/Zoo

TABLE S4. Source type, animal age, sex, and de-sexing status for bedding samples.

Predator	Sex	Desexing status	Age	Source type
Dingo	Female	Entire	Adult	Domestic
Dingo	Female	Entire	Adult	Domestic
Dingo	Male	Desexed	Adult	Domestic
Dingo	Male	Entire	Juvenile	Domestic
Dingo	Male	Entire	Adult	Domestic
Dingo	Male	Entire	Adult	Sanctuary/Zoo
Dingo	Male	Desexed	Adult	Sanctuary/Zoo
Dog	Male	Desexed	Adult	Domestic
Dog	Male	Desexed	Adult	Domestic
Dog	Female	Desexed	Adult	Domestic
Dog	Male	Desexed	Adult	Domestic
Dog	Female	Desexed	Adult	Domestic
Dog	Male	Desexed	Juvenile	Domestic
Dog	Female	Desexed	Adult	Domestic
Cat	Female	Desexed	Adult	Domestic
Cat	Female	Desexed	Adult	Domestic
Cat	Male	Desexed	Adult	Domestic
Cat	Male	Desexed	Adult	Domestic
Cat	Female	Desexed	Adult	Domestic
Cat	Female	Desexed	Adult	Domestic
Tiger quoll	Male	Entire	Adult	Sanctuary/Zoo
Tiger quoll	Female	Entire	Adult	Sanctuary/Zoo
Tiger quoll	Male	Entire	Adult	Sanctuary/Zoo
Tiger quoll	Male	Entire	Adult	Sanctuary/Zoo
Tiger quoll	Male	Entire	Adult	Sanctuary/Zoo
Tiger quoll	Male	Entire	Adult	Sanctuary/Zoo
Tiger quoll	Female	Entire	Adult	Sanctuary/Zoo
Devil	Male	Entire	Adult	Sanctuary/Zoo
Devil	Male	Entire	Adult	Sanctuary/Zoo
Devil	Female	Entire	Adult	Sanctuary/Zoo
Devil	Male	Entire	Adult	Sanctuary/Zoo
Devil	Female	Entire	Adult	Sanctuary/Zoo
Devil	Female	Entire	Adult	Sanctuary/Zoo
Devil	Male	Entire	Adult	Sanctuary/Zoo
Devil	Male	Entire	Adult	Sanctuary/Zoo
Devil	Male	Entire	Adult	Sanctuary/Zoo
Fox	Female	Desexed	Adult	Domestic
Fox	Female	Desexed	Adult	Sanctuary/Zoo
Fox	Female	Entire	Juvenile	Sanctuary/Zoo

Supplementary references

Primer-E Ltd. 2012. Primer-E v.6 and PERMANOVA+. Primer-E Ltd., Plymouth, U.K
